# An In Vitro Study Comparing the Impact and Flexural Strength of Leucitone 199 Denture Base Resin and Conventional Denture Base Resin Enhanced With Glass Fibre Mesh and Polyethylene Fibre Mesh

**DOI:** 10.7759/cureus.45935

**Published:** 2023-09-25

**Authors:** Hileri Vijaysinh Mori, Rajashree Jadhav, Ajay Sabane, Abhijit Patil, Arti Gachake, Bhagyashree Gurunath Kalsekar

**Affiliations:** 1 Department of Prosthodontics and Crown and Bridge, Bharati Vidyapeeth Deemed University, Bharati Vidyapeeth Dental College and Hospital, Pune, IND

**Keywords:** polymethyl methacrylate resin, prosthodontics, polyethylene fiber mesh, denture, leucitone

## Abstract

Purpose

The purpose of this in vitro study was to compare and evaluate the impact and flexural strength of conventional denture base resin reinforced with glass fiber mesh and polyethylene fiber mesh with Leucitone 199 denture base resin (Dentsply Sirona, Charlotte, USA). Leucitone is an expensive denture base material. To come up with a cheaper solution for reinforced denture base resin with superior impact and flexural strength was the goal of this study

Material and methods

The specimens (maxillary denture bases) were fabricated using a standard polyvinylsiloxane mold with conventional heat-cured polymethyl methacrylate resin (ISO 1567:1999). The specimens were divided into three groups (n = 10). Group I specimens, or the control group, were pre-reinforced Leucitone 199. Group II and Group III specimens were reinforced with 3% by weight of glass fibers and polyethylene fibers in weave form, respectively. All the specimens were immersed in water for 1 week before testing. The impact strength was measured with a falling weight impact testing machine. One-way analysis of variance and Tukey’s post hoc test were used for statistical analysis. The flexural strength was measured with the three-point bending test in a universal testing machine.

Results

The highest impact strength values were exhibited by the Leucitone 199 group, followed by the polyethylene fiber mesh group, followed by the glass fibers mesh group. The highest flexural strength was seen in the Leucitone 199 group, followed by the glass fiber mesh group. The least flexural strength was seen in the polyethylene fiber mesh-reinforced group.

Conclusion

Reinforcement of maxillary complete dentures showed a significant increase in impact strength and flexural strength, but not in comparison to pre-reinforced Leucitone 199 dentures. By using pre-impregnated glass and polyethylene fibers in woven form (prepregs), the impact strength of the denture bases can be increased effectively.

## Introduction

Dentures have come a long way since their early beginnings and continue to be an important part of oral care today. With advances in technology, modern denture materials are more durable and comfortable than ever before. Dr. Walter Wright and the Vernon Brothers were the first to introduce acrylic resins in 1937 [1]. By 1940, acrylics accounted for 95% of dentures. The most common polymer for denture bases is polymethyl methacrylate (PMMA). In comparison to many other materials, it excels in every way: in appearance, cost, simplicity of manufacture, and upkeep.

There are a number of issues with it, such as a lingering monomer allergy, problems with mechanical strength, fatigue strength, brittleness on impact, hardness, thermal expansion and contraction, color stability of self-cured resins, porosity, crazing, warping, and so on. mechanical retention is needed, and softness is preferred [2,3].

As a result, there have been numerous new developments in the realm of acrylics to address these shortcomings. To increase strength and other physical qualities, acrylic resins have been reinforced with a variety of components. The difficulties caused by monomer allergy are solved by hypoallergenic resins. To improve its physical and mechanical properties, acrylic resin is being mixed with a wide range of fibers [2-4]. PMMA's poor fatigue tolerance is a big drawback. Seventy percent of maxillary dentures break while being worn,according to studies by Johnston et al. Dentures may break due to impact if they are thrown against a hard surface, or they can break due to fatigue if they are continuously twisted by occlusal pressures [3]. As a result, over time, dentures have a tendency to break while being worn. Maxillary fractures are commonly brought on by a combination of trauma and fatigue (when they accidentally fall on hard surfaces). Maxillary denture midline fractures are the most frequent.

Stainless steel or Co-Cr (cobalt-chromium) alloy wire and plates are frequently employed to reinforce dentures to prevent breakage [2-5]. Test specimens were strengthened using metal wires, glass fiber, carbon, and aramid fibers. The fracture resistance of the PMMA was improved by each metal strengthener. Some fibers had strengthening qualities in addition to being salinized for improved adherence. Polyethylene fibers with ultra-high molecular weight, glass fibers, and polyaromatic polyamide fibers have lately been advised to prevent denture fracture. Despite the fact that polycarbonates and polyamides increase strength, they are costly and technique dependent. Although attempts have been made to alter the chemical body by adding agents such as polyethylene glycol or by copolymerizing with butadiene styrene (rubber), the strength qualities were not noticeably improved [2,4]. Several fibers, including aramid, Kevlar, carbon, glass, yarn, and polyethylene have been used as reinforcement.

Kevlar and carbon fibers are not used due to their toxic nature as well as unattractive color [4-7]. Dentures are reinforced with glass and polyethylene fibers because they are biocompatible and aesthetically pleasing. Since Leucitone 199 denture base resin (Dentsply Sirona, Charlotte, USA) naturally has superior flexural strength and impact than reinforced denture base resin, it's critical to compare the two materials. This research set out to evaluate these dentures against reinforced dentures in terms of their resistance to impact and flexure. Lucitone is an expensive denture base material. To come up with a cheaper solution for reinforced denture base resin with superior impact and flexural strength was the goal of this study.

## Materials and methods

Heat cure denture base resin (DPI, Mumbai, India) was used to create the specimens, which were maxillary full dentures. An elastomeric mold was created using a maxillary denture for the purpose of standardizing specimens. The elastomeric mould was used to create the additional specimens. Leucitone 199-made dentures were utilised as the control group (ISO 1567:1999). As per the manufacturer's instructions, dentures made using traditional denture base resin (DPI) were reinforced with polyethylene fibers and glass in the shape of a weave. There were 30 total maxillary dentures made, 10 for each group.

All 30 samples of the denture base were created using self-curing denture base resin from stone castings made from a standard edentulous rubber mould. With modeling wax, wax occlusal rims of the required size were created. Waxing up and arranging the teeth were completed. After flaking and dewaxing, heat-cure denture base resin was put into the mould. The flask was then treated in an acrylizer with water as directed by the manufacturer. Dentures were deflasked, trimmed, completed, and polished after a slow curing cycle at 70 degrees Celsius for 7 hours and a rise to 100 degrees Celsius for 3 hours. [8,9]

A putty elastomeric mix was adapted to the trial maxillary denture, and after setting, the trial denture was detached to get a putty mold. To create the wax models, a set of teeth from a mould resembling this one (Premadent, Torrance, California) was used, and once it had been positioned in the putty mould, molten modeling wax was poured into the cavity. The duplicate wax denture was removed from the mould after it had cooled for one hour. This procedure produced 30 similar dentures.

Wax models were flasked in bench clamps (Kavo clamps) using regular denture flasks. After that, the flask was dewaxed and let to bench cool.

Control group (Group 1)

Leucitone 199, a heat-cure acrylic denture base material, was prepared in accordance with the guidelines provided by the manufacturer. The two halves of the flasks that were obtained were treated with separating media. In the dough stage, the material was compacted. The cellophane paper was removed after trial packing. The final closing was then carried out by using a hydraulic press to exert pressure of 10,000 N. As was done for the normal specimen, bench curing and acrylization were performed. Ten of these dentures were produced once the specimens were finalized and polished.

Dentures reinforced with glass fiber mesh (Group 2)

The experimental group underwent the identical procedures as the control group up to the application of the separating medium. The flask was then split down the middle and the dough-like combination of heat-cure denture base resin material was poured into both halves with cellophane paper sandwiched in between to complete the trial closure. After the trial was completed, the cellophane paper was taken off, and between the two parts of the flask, both polyethylene fiber (Reliance India Ltd, Nariman Point, Mumbai, India) and glass fiber (Reliance India Ltd, Nariman Point Mumbai, India) mesh were used 3% by weight, covering the whole palatal surface up to the ridge's peak. The hydraulic press was then set to 10,000 N and used to press the flasks. The hydraulic press was then set to 10,000 N and used to press the flasks. Following bench curing, they underwent the same processing as Group I. Recovered dentures were polished and completed.

Dentures reinforced with polyethylene fibers (Group 3)

These samples went through the same processes that the glass fiber group did before being closed. Polyethylene fibers (Reliance India Ltd, Mumbai, India).* *All the steps were followed as for the control group until trial closure. After trial closure, three layers of polyethylene fiber weave (3% by weight) were wetted with monomer and 10 drops of heat-cure monomer and some amount of polymer powder was sprinkled to obtain a prepreg. This prepreg was placed between the two halves of the flasks in two layers. In Group I, the flasks were pressed under the hydraulic press, bench cured, and processed in an acrylizer. The dentures were finished and polished. The dentures in Group II were acrylized, finished, and polished after final closure, the same as those in Group 1.

Testing resistance to impact and bending

The samples were classified as follows (Table 1).

**Table 1:** Sample groups

Group	Sample
Group I	Leucitone 199 maxillary dentures (10 samples)
Group II	Glass fiber mesh-reinforced maxillary dentures (10 samples)
Group III	Polyethylene fiber mesh-reinforced maxillary dentures (10 samples)

Before testing, all the samples were kept in water for seven days. Fifteen samples were put through an impact test using a falling weight machine to determine their impact resistance. Falling weight machine is often exemplified by the Charpy Impact Test Machine (Instron, ISO 148-1, Norwood, US). An impactor and a plastic tube of 1.25 meters in length made up the device. The metal impactor weighed in at 0.836 kg and tapered to a hemisphere with a 50 mm radius. The platform had the tube attached to it. The testing equipment was laid out on a level area. After setting the denture on the platform, we threw the impactor through the plastic tube at various heights. The values were first noted for a height of 0.30 m. If the denture did not break at this height after 40 repetitions, then the height was increased to 0.50 m and testing was done until complete fracture (CF) occurred for all the specimens. Two metrics taken were crack initiation energy (CI) and CF energy.

The impact strength was evaluated using a falling weight impact testing machine on 15 specimens. It consisted of a 1.25 m long plastic tube and an impactor. The plastic tube had three windows to minimize the resistance between the tube and the impactor. The impactor was made out of a metal ball and had a hemisphere end of radius 50 mm and a mass of 0.836 kg. The tube was mounted on a platform. The testing apparatus was placed on a flat surface while testing. The denture was positioned on the platform and the impactor was dropped at different heights through the plastic tube. First, the values were recorded for 0.30 m height. If the denture did not break at this height after 40 repetitions, then the height was increased to 0.50 m and testing was done until complete fracture (CF) occurred for all the specimens.

**Figure 1:**
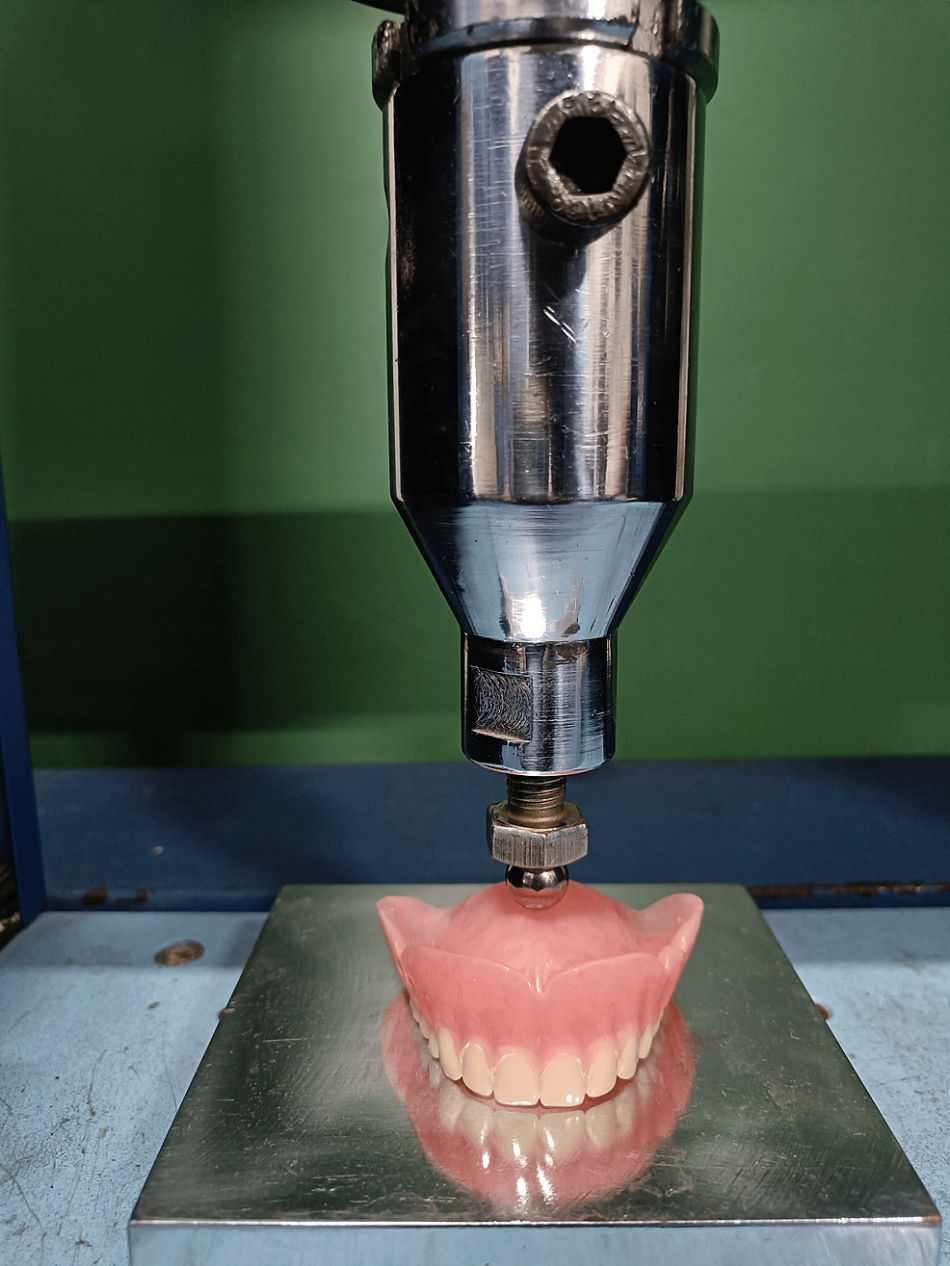
Universal testing machine (please insert a slightly more descriptive figure title)

The three-point bending test was performed on 15 denture specimens in a universal testing device (Computerized software based by ACME Engineers, Pune, India, Model No. UNITEST-10, Accuracy: ±1%, Crosshead speed: 0.75 mm/minute, Distance between supports: 20 mm, Sample Dimension: 25 mm x 2 mm x 2 mm) at the Praj Metallurgical Lab in Pune (Figure 1).

The space between the second molars, which are the last teeth on either side, served as a supporting span and was 42.33 ± 0.2 mm. The load was applied to the palatal fitting surface at the intersection of the palatal midline and the line connecting the centers of the first molars on each side of the denture. At the point of loading, which is located in the central palatal region, a computerized micrometre was used to determine the thickness of the dentures. The typical size was 30.2 mm. The width of the load-bearing area of the dentures was measured as 42 ± 0.2 mm. A sophisticated electronic scale was used to calculate the mass of each denture model. All denture samples were tested for their equivalent flexural strength in MPa using equation. 

The flexural strength of denture base materials is generally evaluated by a three-point bending test on a beam shape sample according to the British standard BS 20795-1:2013 (ISO 20795) using a beam shape sample according to the following equation.

σ = 3Fl / 2bh2

Under static loading conditions, flexural strength was analyzed using one-way ANOVA followed by post hoc Tukey test. All tests used a .05 level of statistical significance. Statistical software SAS for Windows V9.1 (SAS Institute Inc, Cary, USA) was used for data analysis.

## Results

The comparison of the flexural strengths of the three groups is shown in Table 2.

**Table 2:** Comparison of flexural strength meansurement unit: newton (N)

Group	Mean	SD	p value
Lucitone 199	1655.50	184.72	<0.001
Glass fiber mesh	999.10	182.64	
Polyethylene mesh	894.40	269.04	

The glass fiber mesh group and the Lucitone 199 group both displayed the highest flexural strength. Mesh made of polyethylene exhibited the lowest flexural strength. The three groups' overall flexural strengths varied significantly.

Table 3 compares the flexural strength of the three groups pairwise.

**Table 3:** Pairwise comparison of flexural strength meansurement unit: newton (N)

Group	Mean difference	p value
Lucitone 199 vs Glass fiber mesh	656.40	0.001
Lucitone 199 vs Polyethylene mesh	761.10	<0.001
Glass fiber mesh vs Polyethylene mesh	104.70	0.730

Upon comparing Lucitone's flexural strength with that of the glass fiber mesh and polyethylene mesh groups, a considerable difference was seen. Additionally, compared to the polyethylene mesh group, the glass fiber mesh group had a much better flexural strength. 

The comparison of the number of cycles needed to start a denture crack is shown in Tables 4-6.

**Table 4:** Details of the number of cycles and impact strength at crack initiation

Variable	Sample no	Lucitone 199	Glass Fiber Mesh	Polyethylene Mesh
No of cycles at crack initiation	1	58	40	49
	2	56	39	51
	3	57	40	52
	4	59	38	50
	5	55	37	53
	Mean	57	38.8	51
	SD	1.58	1.30	1.58
Impact strength at crack initiation (newton (N))	1	237.59	98.31	200.72
	2	229.40	95.86	208.92
	3	233.49	98.31	213.01
	4	241.69	93.40	204.82
	5	225.30	90.94	217.11
	Mean	233.49	95.36	208.92
	SD	6.48	3.20	6.48

**Table 5:** Details of the number of cycles and impact strength at complete fracture meansurement unit: newton (N)

Variable	Sample no	Lucitone 199	Glass Fiber Mesh	Polyethylene Mesh
No of cycles at complete fracture	1	66	48	58
	2	67	47	58
	3	67	49	56
	4	69	46	59
	5	68	48	55
	Mean	67.40	47.60	57.20
	SD	1.14	1.14	1.64
Impact strength at complete fracture (newton (N))	1	270.36	117.98	237.59
	2	274.46	115.52	237.59
	3	274.46	120.43	229.40
	4	282.65	113.06	241.69
	5	278.56	117.98	225.30
	Mean	276.10	116.99	234.31
	SD	4.67	2.80	6.73

**Table 6:** Comparison of impact strength at crack initiation and complete fracture meansurement unit: newton (N)

Group	Crack initiation		p value	Complete fracture		p value
	Mean	SD		Mean	SD	
Lucitone 199	233.49	6.48	<0.001	276.10	4.67	<0.001
Glass fiber mesh	95.36	3.20		116.99	2.80	
Polyethylene mesh	208.92	6.48		234.31	6.73	

While polyethylene mesh-reinforced dentures produced average results and glass fiber mesh-reinforced dentures cracked after a very small number of cycles, Leucitone 199 material required the most cycles. The number of cycles needed to break the denture varied significantly across the three groups.

The mean of the impact strength for the Lucitone 199, glass fiber mesh, and polyethylene mesh at CI was 233.49 N, 95.36 N, and 208.92 N, and the standard deviations were 6.48 N, 3.20 N, and 6.48 N respectively. Three groups were significantly different from one another in terms of impact strength at CI. Standard deviations were 4.67 N, 2.80 N, and 6.73 N for Lucitone 199, glass fiber mesh, and polyethylene mesh at CF, respectively, with an average impact strength of 276.10 N, 116.99 N, and 234.31 N, respectively. The CF impact strength varied significantly between the three groups.

The impact strengths at CI and CF among the groups are compared pairwise in Table 7.

**Table 7:** Pairwise comparison of impact strength at crack initiation and complete fracture meansurement unit: newton (N)

Pair	Crack initiation				Complete fracture			
	Mean	Mean	Mean difference	p value	Mean	Mean	Mean difference	p value
I vs II	233.49	95.36	138.13	<0.001	276.10	116.99	159.11	<0.001
I vs III	233.49	208.92	24.57	<0.001	276.10	234.31	41.79	<0.001
II vs III	95.36	208.92	113.55	<0.001	116.99	234.31	117.32	<0.001

The test was statistically significant, according to the Tukey's test (P<0.05).

## Discussion

Although there are many advantages to using PMMA, which is often used in the field of prosthodontics to make denture bases, there are also a few drawbacks like low impact strength and low flexural strength [2]. Dentures can break due to impact failure when they are hit/dropped against a hard platform or fatigue failure when the base of the denture is continuously deformed by occlusal forces. Denture fractures involving the maxilla and mandible occur in a ratio of about 2:1 [5-6]. The most typical reasons for fractures include a bad fit and a lack of optimal occlusion. A combination of fatigue and impact failure causes maxillary dentures to break, whereas impact failure causes around 80% of mandibular dentures to break [4].

Many researchers have looked at how stress is distributed inside dentures, employing a range of techniques and theoretical models, when they are accidentally thrown upon a hard surface. They suggested that the polished surfaces of the incisors and labial aspect produce tensile stresses that cause bending deformation of the maxillary dentures [8-10]. A weak spot that could act as a stress raiser and lead to fractures in the midline of upper jaw dentures is the upper incisal notch. The incisive papilla, or the region from the lingual to the anteriors, is the most severely stressed [11,12].

Compressive stresses exist in the supporting tissues in the maxillary base, while tensile stresses are present elsewhere, according to a thorough photoelastic stress analysis [13]. Tissues were compressed at their surfaces; forces were greater behind and on the edges of the teeth than they were around the taste buds. Functional loading will have an impact on the stress distribution, with the upper denture midline experiencing the lowest stress. Maximum stresses are found around molars in the centric occlusion, with stresses increasing from the anteriors to the molars over the ridge [14]. Although this biting power is lower than normal, patients with complete dentures can bite with a maximum force of up to 200 N** **(ISO 14457) around molars and up to 80 N around incisors [15].

Flexural fatigue is the result of the denture base's cyclic deformation during use, which can lead to midline fracture. Any element that increases the denture base's deformation or changes the stress distribution within it puts the denture at risk of breaking. A stress raiser or a point of localized stress must be present for a crack to start and spread, which results in fracture. The palatal midline acts as a fulcrum, midline palatal fractures of acrylic dentures happen, and upward displacement of the dentures causes the denture to break into two parts [14].

As a result, many attempts have been made to improve the strength characteristics of acrylic denture bases. In one of the attempts, it has been tried to change the chemical structure by copolymerizing with rubber material or by adding agents such as polyethylene glycol di-methacrylate. These techniques might not, however, result in a material's strength increasing as much. The foundation of acrylic resin dentures has been strengthened using a variety of techniques. The various fibers employed have included carbon, aramid, glass, and polyethylene fibers. Metal inserts have also been employed in the forms of wires, meshes, and thin plates. Carbon and aramid fibers aren't utilized because of their toxicity and lack of aesthetic appeal [2-4]. Stiff, strong, biocompatible, seeming white and transparent, and absorbing very little water, glass, and polyethylene fibers are successfully employed for reinforcing [2]. The maxillary dentures in the current investigation are reinforced with glass and polyethylene fibers.

There are two approaches to reinforcing the denture foundation: either the entire base can be strengthened, or strengthening can be done at the denture's weak spot. According to research, the midline curve of the denture base changes throughout mastication and swallowing procedures, along with the flanges' slight expansion and compression [15]. As a result, the complete denture base should be strengthened with fibers that are orientated laterally; strengthening may be performed by focusing on the horizontally aligned parallel fibers. In the current investigation, the reinforcement was done in the complete palatal surface extending up to the crest of the ridge for the maxillary dentures.

Fibre reinforcing may be accomplished with individual fibers in either a continuous parallel or chopped form or with woven mats [16]. The woven reinforcing is particularly good at controlling the spread of the crack. The resin should fully permeate the weave for optimal reinforcement, making sure there are no voids at the fiber/resin interface [17-20]. Heat-cure acrylic resin's dough-like viscosity may make it difficult for the resin to seep into the weave. The fiber inserts may be wetted with PMMA monomer material or a thin PMMA slurry to generate a prepreg before the final closure. Researchers have established that the ideal percentage of fiber incorporation is required for improved strength. Good impact strength is obtained when fibers are included at a weight percentage of around 3% [2]. Dentures absorb saliva and water while in use. A linear change in diameter from flange to flange of roughly 0.3% is possible during the first three months of immersion. While long-term water sorption will reduce the acrylic resin's fatigue resistance, this may have an impact on how the material deforms under stress. Due to the hydrophobic nature of fibers, decreased water absorption is correlated with increased fiber content. The mechanical characteristics and interface strength of dental resins reinforced with fibers are typically not significantly affected by water immersion. According to studies, denture bases containing 2-7% by weight of fiber will not have an impact on the acrylic resin's ability to maintain accurate dimensions [3]. In this investigation, prepregs made of woven glass and polyethylene fibers accounted for 3% of the reinforcing weight, and the specimens were kept submerged in water for a week.

The most typical substance utilized for detachable prosthetic and orthodontic items is acrylic resin. Many brands have created high-impact resins to solve the denture's intrinsic shortcomings. This includes a lack of durability due to the denture's susceptibility to damage from impacts and flexing. High impact denture base materials are now in broad usage, thus it's important for physicians to understand their characteristics [21-24]. 

The flexural strength of pre-reinforced Leucitone 199 against conventional denture base resin reinforced with nylon and glass fibers was tested in a study by Kannaiyan K et al. [24]. The specimen size used in this experiment showed statistical significance when the values for flexural strength were contrasted to the unreinforced large impact of denture base resin (Leucitone 199). However, maxillary dentures were used as a specimen in the current study. In this study, Leucitone 199's flexural and impact strength were shown to be higher than those of glass fiber- and polyethylene fiber-reinforced denture specimens.

In order to compare the mechanical qualities of reinforced PMMA with a maxillary denture as a specimen to the commercially successful pre-reinforced Leucitone 199 denture base resin, Leucitone was chosen as the control group for this investigation. This study therefore has significant clinical relevance and value. There has been much study into the impact strength of acrylic resin that has been reinforced with a wide variety of fibers and metal implants [25]. Acrylate resin blocks cut into squares were used as test specimens everywhere. In these experiments, flexing beam impact techniques were utilized, such as a Charpy-type pendulum impact tester or a Zwick pendulum impact tester, but a falling weight impact testing equipment may also be used to mimic real-world clinical conditions in which a denture would be worn. In the present investigation, maxillary denture impact resistance was evaluated using a falling weight impact testing equipment. PMMA denture base resin's impact strength may be improved by fiber reinforcement. The fiber's impact resistance has been greatly enhanced by the treatment given to its surface [20]. In this investigation, the polyethylene fiber group outperformed silane-treated glass fibers in terms of impact strength. 

## Conclusions

Leucitone 199 maxillary dentures outperformed glass fiber mesh-reinforced and polyethylene fiber mesh-reinforced dentures in impact and flexural strength. Denture base polymer strengthened with polyethylene fibers and glass fiber mesh exhibited similar impact strength. Pre-impregnated glass and polyethylene fibers in woven mesh form (prepregs) can strengthen denture bases, although it is not significantly stronger than Leucitone 199's impact strength. Leucitone 199 had the strongest impact strength. Polyethylene denture bases had less flexure than polymer bonded with glass fiber mesh.
